# RETRACTION: Activation of AdipoR1 with rCTRP9 Preserves BBB Integrity through the APPL1/AMPK/Nrf2 Signaling Pathway in ICH Mice

**DOI:** 10.1155/omcl/9852076

**Published:** 2026-03-16

**Authors:** Oxidative Medicine and Cellular Longevity

**RETRACTION**: W. Zhao, F. Kong, X. Gong, Z. Guo, L. Zhao, and S. Wang, “Activation of AdipoR1 with rCTRP9 Preserves BBB Integrity through the APPL1/AMPK/Nrf2 Signaling Pathway in ICH Mice,” *Oxidative Medicine and Cellular Longevity* 2021, no. 1 (2021): 1–14, https://doi.org/10.1155/2021/2801263.

The above article, published online on 08 December 2021 in Wiley Online Library (wileyonlinelibrary.com), has been retracted by agreement between the journal Editor‐in‐Chief, Jeannette Vasquez‐Vivar; and John Wiley & Sons Ltd. The retraction has been agreed due to concerns raised with Figures 1 and 4, initially on PubPeer [1]. Specifically:

• The Actin bands shown in Figure 1a appear identical to the ß‐Actin bands shown in Figure 7d of a different article, with one of the same authors [2]
• The CTRP9 bands shown in Figure 4a appear identical when stretched to the AdipoR1 bands shown in Figure 1b of [2]

The authors contacted the Journal to correct the Figures, however were unable to provide the original images. After assessment by the Chief Editor, the results and conclusions were deemed unreliable and therefore the article must be retracted.

The authors did not respond when informed of the retraction.
